# Associations Between Ending Supplemental Nutrition Assistance Program Emergency Allotments and Food Insufficiency

**DOI:** 10.1001/jamahealthforum.2023.2511

**Published:** 2023-08-11

**Authors:** Aaron Richterman, Christina A. Roberto, Harsha Thirumurthy

**Affiliations:** 1 Division of Infectious Diseases, Hospital of the University of Pennsylvania, Philadelphia; 2 Leonard Davis Institute for Health Economics, University of Pennsylvania, Philadelphia; 3 Department of Medical Ethics and Health Policy, University of Pennsylvania, Philadelphia

## Abstract

This cross-sectional study evaluates associations between changes in Supplemental Nutrition Assistance Program emergency allotments and food insufficiency, a severe form of food insecurity characterized by recent food inadequacy.

## Introduction

Food insecurity—a lack of stable access to food in adequate quantity or quality—can negatively influence health through multiple pathways.^1^ The Supplemental Nutrition Assistance Program (SNAP) distributes cash-like benefits to impoverished households in the US to buy food, with an average benefit size of approximately $240 a month per household before the COVID-19 pandemic. SNAP’s benefit size (coupled with challenges in accessing SNAP) has historically limited the program’s ability to fully address food insecurity, which continues to affect roughly 10% of US households.^2^ During the COVID-19 pandemic, SNAP emergency allotments (EAs) provided temporary increases to households’ benefit size—either to the maximum or by $95, whichever was greater. These EAs were the largest-ever increases in SNAP benefit amounts. The EAs ended in March 2023, but 18 states discontinued them even earlier when ending their state’s public health emergency (eTable 1 in Supplement 1). This state-level variation in SNAP benefit size allowed us to evaluate associations between changes in SNAP benefit amounts and *food insufficiency*, a severe form of food insecurity characterized by recent food inadequacy.

## Methods

In this cross-sectional study, we obtained repeated data from the US Census Bureau’s Household Pulse Survey, which recurs every 2 to 4 weeks and generates nationally representative and state-representative estimates, from August 2020 to February 2023.^3^ We classified respondents by month and year of survey wave (eTable 2 in Supplement 1) and by state. The study exposure was whether EAs had ended at the state-month level. The study outcomes were household food insufficiency (sometimes/often not enough food to eat in the last 7 days), overall and among children. We used 2-stage linear difference-in-differences models including state and month fixed effects,^4^ with further adjustment for age, household size, race and ethnicity, education, income, employment and receipt of free food (last 7 days), calendar month, and government-reported state-month-level proportion of individuals covered by SNAP,^5^ with state cluster-robust standard errors. Primary analyses included all survey respondents (because SNAP receipt is underreported by respondents). Secondary analyses were restricted to respondents reporting current household SNAP receipt, as they would benefit the most from EAs, and to respondents not reporting SNAP receipt with household income of more than $100 000 (ie, likely not SNAP-eligible) as a falsification test. We evaluated dynamic effect estimates by creating binary indicators for each month before and after EAs ended. We applied survey weights to descriptive/comparative statistics,^6^ with significance at 2-sided *P* < .05. This study met the STROBE reporting guideline. The University of Pennsylvania institutional review board waived ethical review. Informed consent was waived because all data were deidentified. We conducted analyses using Stata statistical software, version 17 (StataCorp).

## Results

There were 3 001 647 survey respondents during the study period, of whom 28% lived in states that ended EAs early, and 12% reported their household received SNAP. The mean (SD) age was 48 (16) years, and 51% identified as female. Approximately 5% identified as Asian, 17% Hispanic, 11% non-Hispanic Black, and 62% non-Hispanic White (self-reported). The mean (SD) state-month-level proportion of households covered by SNAP was 0.18 (0.06),^5^ indicating that SNAP receipt was underreported by approximately one-third in the survey. Adjusted analyses showed that ending EAs was associated with significantly higher overall and child food insufficiency, with point estimates translating to 5% and 6% relative increases in prevalence, respectively (Table). Associations were most pronounced among reported SNAP recipients, who experienced approximately a 21% relative increase in both food insufficiency and child food insufficiency. There were no significant changes among respondents not reporting SNAP receipt with household income of more than $100 000. Observed associations persisted out to 15 months, and there was no evidence of differential trends in outcomes before EAs ended (Figure). When comparing respondents during the 2 months before and after EAs ended, small differences were found in recent employment (57% vs 55%; *P* = .01) and state-month-level per-household SNAP coverage (0.183 vs 0.175; *P* < .001), implying findings were unlikely a result of changes in economic conditions or SNAP coverage.

**Table.** Unadjusted and Adjusted Associations Between Ending SNAP Emergency Allotments and Household Food Insufficiency^a^^,^^b^

Outcomes	Emergency allotments, No. (%)	No emergency allotments, No. (%)^c^	Percentage point change after ending emergency allotments (95% CI)	
	Outcome population prevalence	Outcome population prevalence	State and month fixed effects	Additional adjustment^d^
**All respondents**				
Food insufficiency	2 481 954 (10)	227 030 (12)	0.75 (0.55 to 0.96)	0.54 (0.38 to 0.69)
Child food insufficiency	2 390 378 (3)	216 359 (3)	0.29 (0.22 to 0.36)	0.18 (0.08 to 0.28)
**Reported receiving SNAP**				
Food insufficiency	183 448 (25)	15 319 (31)	5.92 (4.56 to 7.28)	5.39 (4.42 to 6.36)
Child food insufficiency	163 165 (10)	13 014 (12)	2.33 (1.63 to 3.03)	2.16 (1.51 to 2.79)
**Reported not receiving SNAP and household income ≥$100 000**				
Food insufficiency	826 973 (1)	63 604 (2)	0.07 (−0.03 to 0.16)	0.07 (−0.02 to 0.18)
Child food insufficiency	823 381 (<1)	63 306 (<1)	0.02 (−0.03 to 0.08)	0.01 (−0.04 to 0.07)

Abbreviation: SNAP, Supplemental Nutrition Assistance Program.

^a^ Food insufficiency is defined as sometimes/often not enough food to eat in the last 7 days, overall and among children.

^b^ Unweighted sample sizes and weighted outcome population prevalence for each group are also shown.

^c^ Observations from the 18 states that ended emergency allotments before March 2023 (see eTable 1 in Supplement 1).

^d^ Adjusted for individual/household-level covariates (age, number of household members, race and ethnicity, educational attainment, income category [including missing as a category], employment in the last 7 days, and receipt of free food within the last 7 days), calendar month, and number of SNAP participants per capita (state-month-level).

**Figure.**
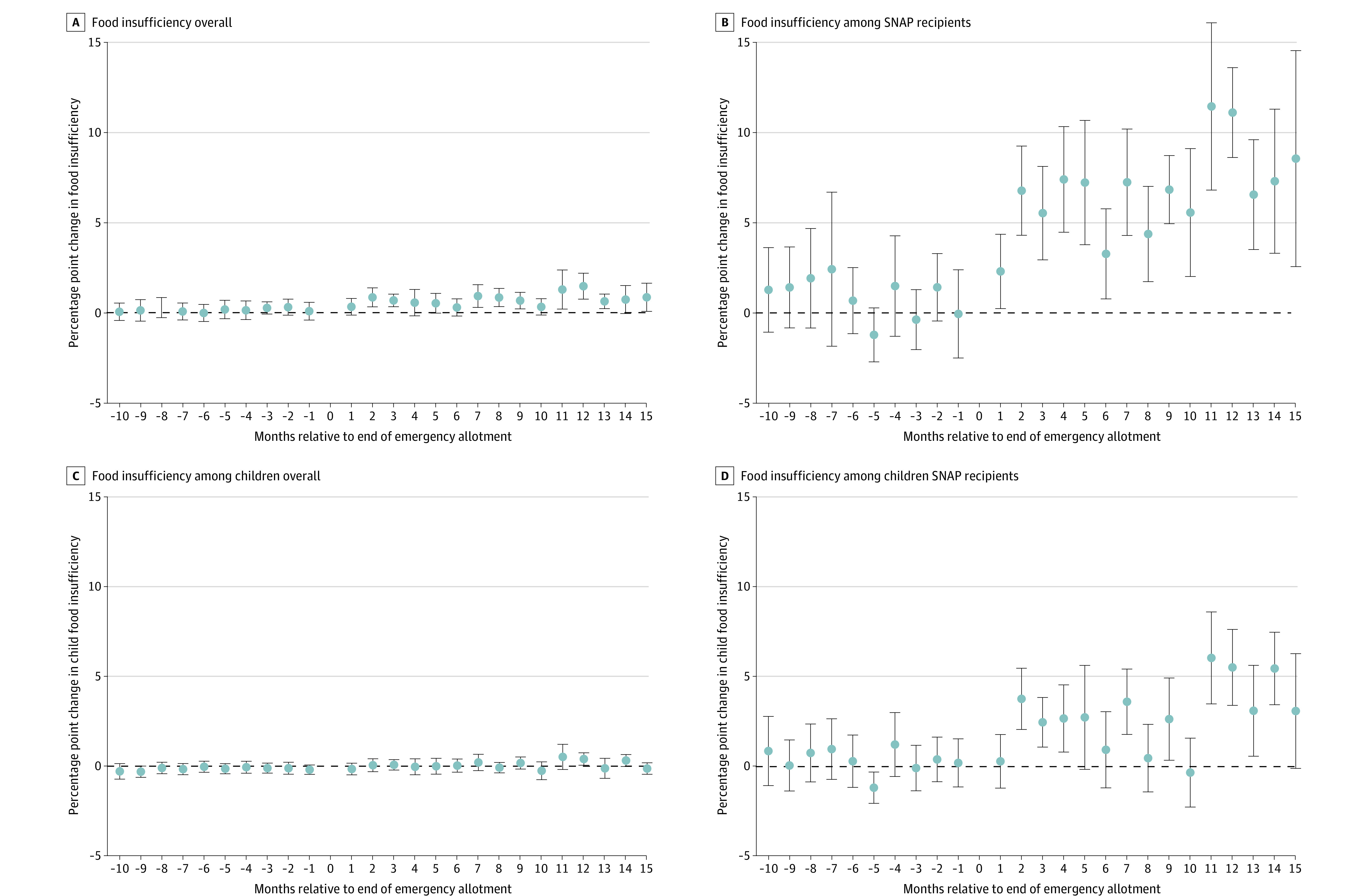
Changes in Food Insufficiency Over Time After Ending Supplemental Nutrition Assistance Program (SNAP) Emergency Allotments Fully adjusted estimates of overall associations between ending SNAP emergency allotments with percentage point changes in outcomes: A, overall food insufficiency among all respondents; B, overall food insufficiency among SNAP recipients; C, child food insufficiency among all respondents; and D, child food insufficiency among SNAP recipients. The dashed line represents no change in food insufficiency. The error bars represent 95% CIs.

## Discussion

In this cross-sectional study, ending SNAP EAs was associated with persistent negative changes in food insufficiency. Study limitations included lack of a more comprehensive food security measure. Increasing SNAP benefit sizes should be considered to reduce food insecurity and improve health.

## Notes

Supplement 1. **eTable 1.** States that ended SNAP emergency allotments prior to March 2023 **eTable 2.** Household Pulse Survey waves included in the study, along with month and year that each wave was classified

Supplement 2.
Data Sharing Statement

## References

[1] Weiser SD, Palar K, Hatcher AM, Young S, Frongillo EA, Laraia B. Food Insecurity and Health: A Conceptual Framework. In: Ivers LC, ed. Food Insecurity and Public Health. CRC Press; 2015:23-41. doi:10.1201/b18451-3

[2] Gundersen C, Seligman H. How can we fully realize SNAP’s health benefits? N Engl J Med. 2022;386(15):1389-1391. doi:10.1056/NEJMp2200306 35417933

[3] US Census Bureau. Household Pulse Survey technical documentation. Updated March 2023. Accessed March 2023. https://www.census.gov/programs-surveys/household-pulse-survey/technical-documentation.html

[4] Gardner J. Two-stage differences in differences. arXiv. Preprint posted online July 13, 2022. doi:10.48550/arXiv.2207.05943

[5] US Department of Agriculture. SNAP data tables. Updated March 2023. Accessed March 2023. https://www.fns.usda.gov/pd/supplemental-nutrition-assistance-program-snap

[6] Solon G, Haider SJ, Wooldridge JM. What are we weighting for? J Hum Resour. 2015;50(2):301-316. doi:10.3368/jhr.50.2.301

