# Supplementary material for: Associations Between Ending Supplemental Nutrition Assistance Program Emergency Allotments and Food Insufficiency
Source: JAMA Health Forum. 2023 Aug 11;4(8):e232511. doi: 10.1001/jamahealthforum.2023.2511 (PMC10422192; doi:10.1001/jamahealthforum.2023.2511)
Supplement: Supplement 1. — eTable 1. States that ended SNAP emergency allotments prior to March 2023 eTable 2. Household Pulse Survey waves included in the study, along with month and year that each wave was classified [file jamahealthforum-e232511-s001.pdf]

Supplemental Online Content

Richterman A, Roberto CA, Thirumurthy H. Associations between ending Supplemental Nutrition Assistance Program emergency allotments and food insufficiency. *JAMA Health Forum.* 2023;4(8):e232511. doi:10.1001/jamahealthforum.2023.2511

eTable 1. States that ended SNAP emergency allotments prior to March 2023

eTable 2. Household Pulse Survey waves included in the study, along with month and year that each wave was classified

This supplemental material has been provided by the authors to give readers additional information about their work.

© 2023 Richterman A et al. *JAMA Health Forum.*

eTable 1. States that ended SNAP Emergency Allotments prior to March 2023

State	Month EAs ended
Idaho	3/2021
North Dakota	5/2021
Arkansas	6/2021
Montana	7/2021
Florida	7/2021
Nebraska	7/2021
South Dakota	7/2021
Missouri	8/2021
Tennessee	12/2021
Mississippi	12/2021
Iowa	3/2022
Wyoming	4/2022
Kentucky	4/2022
Arizona	4/2022
Georgia	5/2022
Indiana	5/2022
Alaska	8/2022
South Carolina	1/2023
All other states	3/2023

© 2023 Richterman A et al. *JAMA Health Forum.*

eTable 2. Household Pulse Survey waves included in the study, along with month and year that each wave was classified. If a wave occurred during two months, we chose the month with a greater number of survey days.

Survey Wave	Start Date	End Date	Month, Year Classification
13	8/18/20	8/31/20	Aug 2020
14	9/2/20	9/14/20	Sep 2020
15	9/16/20	9/28/20	Sep 2020
16	9/30/20	10/12/20	Oct 2020
17	10/14/20	10/26/20	Oct 2020
18	10/28/20	11/9/20	Nov 2020
19	11/11/20	11/23/20	Nov 2020
20	11/25/20	12/7/20	Dec 2020
21	12/9/20	12/21/20	Dec 2020
22	1/6/21	1/18/21	Jan 2021
23	1/20/21	2/1/21	Jan 2021
24	2/3/21	2/15/21	Feb 2021
25	2/17/21	3/1/21	Feb 2021
26	3/3/21	3/15/21	Mar 2021
27	3/17/21	3/29/21	Mar 2021
28	4/14/21	4/26/21	Apr 2021
29	4/28/21	5/10/21	May 2021
30	5/12/21	5/24/21	May 2021
31	5/26/21	6/7/21	Jun 2021
32	6/9/21	6/21/21	Jun 2021
33	6/23/21	7/5/21	Jun 2021
34	7/21/21	8/2/21	Jul 2021
35	8/4/21	8/16/21	Aug 2021
36	8/18/21	8/30/21	Aug 2021
37	9/1/21	9/13/21	Sep 2021
38	9/15/21	9/27/21	Sep 2021
39	9/29/21	10/11/21	Oct 2021
40	12/1/21	12/13/21	Dec 2021
41	12/29/21	1/10/22	Jan 2022
42	1/26/22	2/7/22	Feb 2022
43	3/2/22	3/14/22	Mar 2022
44	3/30/22	4/11/22	Apr 2022
45	4/27/22	5/9/22	May 2022
46	6/1/22	6/13/22	Jun 2022
47	6/29/22	7/11/22	Jul 2022
48	7/27/22	8/8/22	Aug 2022
49	9/14/22	9/28/22	Sep 2022

© 2023 Richterman A et al. *JAMA Health Forum.*

50	10/5/22	10/17/22	Oct 2022
51	11/2/22	11/14/22	Nov 2022
52	12/9/22	12/19/22	Dec 2022
53	1/4/23	1/16/23	Jan 2023
54	2/1/23	2/13/23	Feb 2023

© 2023 Richterman A et al. *JAMA Health Forum.*
