# Supplementary material for: Associations Between Ending Supplemental Nutrition Assistance Program Emergency Allotments and Food Insufficiency
Source: JAMA Health Forum. 2023 Aug 11;4(8):e232511. doi: 10.1001/jamahealthforum.2023.2511 (PMC10422192; doi:10.1001/jamahealthforum.2023.2511)
Supplement: Supplement 2. — Data Sharing Statement [file jamahealthforum-e232511-s002.pdf]

Data Sharing Statement

Richterman. Associations Between Ending Supplemental Nutrition Assistance Program Emergency Allotments and Food Insufficiency. *JAMA Health Forum*. Published August 11, 2023. doi:10.1001/jamahealthforum.2023.2511

Data
Data available: Yes
Data types: Deidentified participant data
How to access data: All data are publicly available from the US Census Bureau (https://www.census.gov/programs-surveys/household-pulse-survey/data.html)
When available: With publication

Supporting Documents
Document types: None

Additional Information
Who can access the data: Anyone who wants the data can freely download it from the US Census Bureau.
Types of analyses: Any analyses.
Mechanisms of data availability: Without investigator support, publicly availably from the US Census Bureau website.
